# CATHe: detection of remote homologues for CATH superfamilies using embeddings from protein language models

**DOI:** 10.1093/bioinformatics/btad029

**Published:** 2023-01-17

**Authors:** Vamsi Nallapareddy, Nicola Bordin, Ian Sillitoe, Michael Heinzinger, Maria Littmann, Vaishali P Waman, Neeladri Sen, Burkhard Rost, Christine Orengo

**Affiliations:** Institute of Structural and Molecular Biology, University College London, London WC1E 6BT, UK; Institute of Structural and Molecular Biology, University College London, London WC1E 6BT, UK; Institute of Structural and Molecular Biology, University College London, London WC1E 6BT, UK; Department of Informatics, Bioinformatics and Computational Biology—i12, Technical University of Munich (TUM), Garching/Munich 85748, Germany; Department of Informatics, Bioinformatics and Computational Biology—i12, Technical University of Munich (TUM), Garching/Munich 85748, Germany; Institute of Structural and Molecular Biology, University College London, London WC1E 6BT, UK; Institute of Structural and Molecular Biology, University College London, London WC1E 6BT, UK; Department of Informatics, Bioinformatics and Computational Biology—i12, Technical University of Munich (TUM), Garching/Munich 85748, Germany; Institute for Advanced Study (TUM-IAS), Garching/Munich 85748, Germany; TUM School of Life Sciences Weihenstephan (WZW) 85354, Germany; Institute of Structural and Molecular Biology, University College London, London WC1E 6BT, UK

## Abstract

**Motivation:**

CATH is a protein domain classification resource that exploits an automated workflow of structure and sequence comparison alongside expert manual curation to construct a hierarchical classification of evolutionary and structural relationships. The aim of this study was to develop algorithms for detecting remote homologues missed by state-of-the-art hidden Markov model (HMM)-based approaches. The method developed (CATHe) combines a neural network with sequence representations obtained from protein language models. It was assessed using a dataset of remote homologues having less than 20% sequence identity to any domain in the training set.

**Results:**

The CATHe models trained on 1773 largest and 50 largest CATH superfamilies had an accuracy of 85.6 ± 0.4% and 98.2 ± 0.3%, respectively. As a further test of the power of CATHe to detect more remote homologues missed by HMMs derived from CATH domains, we used a dataset consisting of protein domains that had annotations in Pfam, but not in CATH. By using highly reliable CATHe predictions (expected error rate <0.5%), we were able to provide CATH annotations for 4.62 million Pfam domains. For a subset of these domains from *Homo sapiens*, we structurally validated 90.86% of the predictions by comparing their corresponding AlphaFold2 structures with structures from the CATH superfamilies to which they were assigned.

**Availability and implementation:**

The code for the developed models is available on https://github.com/vam-sin/CATHe, and the datasets developed in this study can be accessed on https://zenodo.org/record/6327572.

**Supplementary information:**

Supplementary data are available at *Bioinformatics* online.

## 1 Introduction

The CATH database (www.cathdb.info) (Sillitoe *et al.*, 2021) classifies protein domain structures into superfamilies when there is strong evidence that domains share a common evolutionary ancestor. The resource was established in 1994 and currently comprises over 500 000 domains from experimental structures in the Worldwide Protein Data Bank (wwPDB) (wwPDB consortium, 2019), classified into 5481 CATH superfamilies. The classification protocol exploits a number of computational methods to provide evidence for evolutionary relationships including structure-based [SSAP (Taylor and Orengo, 1989) and CATHEDRAL (Redfern *et al.*, 2007)] and sequence-based comparison tools [HMMER (Mistry *et al.*, 2013), PRC (Madera, 2008), HHsearch (Steinegger *et al.*, 2019a) and MMseqs2 (Steinegger and Söding, 2017)]. Since experimental structural data are only available for a small (<1%) fraction of known protein sequences, sequence-based tools are used to detect CATH homologues from large protein sequence resources, such as UniProt (UniProt Consortium, 2015) and ENSEMBL (Aken *et al.*, 2016). In the latest version of CATH [version 4.3 (Sillitoe *et al.*, 2021)], hidden Markov models (HMMs) derived from CATH structural superfamilies (i.e. CATH-HMMs) were used to predict CATH domain locations and superfamilies for over 150 million protein domain sequences (Lewis *et al.*, 2018).

HMM–HMM strategies like HHblits (Remmert *et al.*, 2012) have proven powerful in detecting very remote homologues. However, methods that improve on HHblits in sensitivity and speed would allow CATH and other related resources to keep pace with the expansion in the protein sequence repositories, including MGnify (Mitchell *et al.*, 2020), and the Big Fantastic Database (Steinegger and Söding, 2018; Steinegger *et al.*, 2019b) which are about 20-fold, and 11-fold larger than UniProt, respectively. In the future, high-quality 3D models for UniProt sequences [e.g. from AlphaFold2 (AF2) (Jumper *et al.*, 2021, p. 2)] could be scanned against CATH domain structures to detect homologues. However, currently the low coverage of structural data in CATH compared with sequence data means that many relationships would be missed. Furthermore, the most sensitive structure comparison algorithms tend to be much slower than sequence-based methods.

Recently, numerical representations (embeddings) of protein sequences obtained from protein language models (pLMs) have gained a lot of interest as important input features for classification of protein superfamilies. Language models (LMs), commonly used in the domain of natural language processing (NLP), were adapted to the protein space. Instead of being trained on natural language, they are trained on large protein sequence sets from UniProt. The so-called self-supervised pre-training (a special form of unsupervised training) of these pLMs allows them to readily derive benefits from large unlabelled data as they are only trained on reconstructing corrupted input tokens (words/amino acids) from non-corrupted sequence context (sentence/protein sequence). In a second step, the learned information can be transferred to downstream tasks (transfer learning).

Bepler and Berger (2021) used sequence information extracted from pLMs in conjunction with structural information to predict SCOP families derived from the ASTRAL benchmark dataset (Brenner *et al.*, 2000). Their proposed model, the MT-LSTM, achieved an accuracy of 96.19%. However, while most studies use a 25% sequence identity threshold for removing redundancy between training and test sets, they used a more redundant dataset by applying a sequence redundancy filter of 40%. They found that including structural data led to a much better organization of the proteins in the embedding space when compared with the use of sequence information alone. An important conclusion from this study was the fact that pLMs had the power to capture evolutionary information from sequence alone. More recently, the ProtENN (Bileschi *et al.*, 2022) method used an ensemble deep learning framework that generated protein sequence embeddings to classify protein domains into Pfam (Mistry *et al.*, 2021) families. ProtENN applied an ensemble built from 13 variations of the ProtCNN models which were developed using the ResNet (He *et al.*, 2016) architecture. ProtENN achieved an accuracy of 87.8% when the sequence identity between testing and training sets was set to be less than 25%.

Other approaches that seek to improve on HMM-based strategies for protein evolutionary classification but do not use pLMs include DeepFam (Seo *et al.*, 2018), which was developed to classify protein sequences from the Clusters of Orthologous Groups (COG) of proteins database and the G Protein-Coupled Receptor (GPCR) dataset. DeepFam did not employ a pLM to generate protein sequence embeddings but instead used one-hot encodings of the sequence as input for a neural network model; furthermore, there was no application of a sequence redundancy filter on their dataset. DeepFam attained a prediction accuracy of 97.17% on the family level on the GPCR dataset, and 95.4% on the COG dataset (with protein families having at least 500 sequences). DeepNOG (Feldbauer *et al.*, 2020) employed a method similar to DeepFam to classify sequences from COG and eggNOG5 databases.

In this study, we employed the ProtT5 (Elnaggar *et al.*, 2022) pLM to recognize very remote homologues (i.e. less than 20% sequence identity). We chose ProtT5 pLM as it had previously been successfully employed in other related tasks such as prediction of protein structure (Weißenow *et al.*, 2021), function (Littmann *et al.*, 2021) and prediction of single amino acid variant effect (Meier *et al.*, 2021). Our new approach (CATHe; short for CATH embeddings) uses embeddings from ProtT5 as input to train machine learning models to classify protein sequences into CATH superfamilies. To make sure that our models were capable of detecting very remote homologues in these superfamilies, they were trained on non-redundant homologues with at most 20% sequence identity. We have therefore used a more stringent test set than the approaches mentioned above for protein family classification (i.e. DeepFam and deepNOG).

The performance of the proposed CATHe model was measured on two sets: one consisting of the largest 1773 superfamilies in CATH and the other of the top largest 50 superfamilies. The model achieved an accuracy of 85.6% on the former and 98.2% on the latter set of more highly populated superfamilies. Furthermore, CATHe was able to predict CATH superfamily annotations for 4.62 million Pfam domains that could not be mapped to CATH superfamilies using HMM models. For a subset of these Pfam domains in *Homo sapiens*, that had good quality AF2 models, we were able to structurally validate 91% of the CATHe predictions using the structure comparison method SSAP (Taylor and Orengo, 1989).

## 2 Materials and methods

### 2.1 TOP 1773 SUPERFAMILIES dataset

The aim of this study is to develop a deep learning tool to detect remote homologues for CATH superfamilies. In order to achieve this, it is necessary to generate a dataset from which the classifiers can learn. We made sure that the testing and validation sets of the dataset consisted only of sequences from the Protein Data Bank (PDB) (wwPDB consortium, 2019), whereas the training set contained sequences from CATH-Gene3D (v4.3) (Lewis *et al.*, 2018). CATH-Gene3D contains all the UniProt sequences which can be assigned to CATH superfamilies via scanning against CATH-HMMs (Sillitoe *et al.*, 2021) (which uses an *e*-value of 1*e*−3). The following steps were conducted to generate the dataset for this task:

- Cluster the sequence domains present in CATH from the PDB using MMseqs2 (Steinegger and Söding, 2017) with a 20% sequence identity filter. To gain an in-depth understanding of the data processing using MMseqs2, see Supplementary Section S2.1.
- Using the MMseqs2 output from step (a), choose those superfamilies that have at least two PDB sequences so that they can be split into testing and validation sets. The remaining superfamilies are used to create a ‘mixed-bag’ class summarizing all ‘other’ superfamilies classes.
- Build the training set using all the sequence domains from CATH-Gene3D for all the superfamilies decided in step (b) (i.e. superfamilies that have at least two PDB S20 sequences and ‘other’ superfamilies).
- Use MMseqs2 to reduce the sequence identity between training and the other two (testing and validation) sets to less than 20%.
- As a further check, use BLAST (Altschul *et al.*, 1990) to remove homologous sequences, at 20% sequence identity, between the three sets.
- Randomly under-sample the ‘other’ superfamilies class to reduce the class imbalance in the dataset.
- Generate embeddings for the protein sequences using the pLMs ProtBERT and ProtT5 (Elnaggar et al., 2022). To understand more about the pLMs and the embedding generation process, refer to Supplementary Section S2.1.

After step (a), we identified 1773 CATH superfamilies, having at least two non-redundant PDB sequences. We set the threshold at two sequences because that is the minimum number of sequences that we need in order to split the resultant set into testing and validation sets. The rest of the superfamilies in CATH (3456 superfamilies) were used to make the ‘other’ superfamilies class. The 1773 superfamilies had a total of 82 720 883 domain sequences in the CATH database (which is 87.6% of the CATH v4.3 database) before being processed. The number of sequences in each of the three sets after processing is given in Table 1. This dataset is referred to as the ‘TOP 1773 SUPERFAMILIES’ dataset.

**Table 1.** Description of the training, validation and testing sets for the two datasets generated by processing the data from the CATH database

Dataset	Number of training sequences	Number of validation sequences	Number of testing sequences
TOP 1773 SUPERFAMILIES	1 039 135	6863	6862
TOP 50 SUPERFAMILIES	528 863	1948	1946

### 2.2 TOP 50 SUPERFAMILIES dataset

To investigate more closely the performance on large (i.e. with high numbers of predicted domains) and highly diverse superfamilies, a subset of the TOP 1773 SUPERFAMILIES dataset that summarizes the 50 largest CATH superfamilies was created. This set holds a total of 39 625 167 domain sequences in the CATH database (37.32% of the CATH domain sequences) and will be dubbed TOP 50 SUPERFAMILIES throughout the text.

The number of sequences in training, testing and validation sets after processing is given in Table 1.

### 2.3 Models

Traditional bioinformatics techniques, as well as advanced machine learning methods, were used to develop classifiers to detect remote homologues. The various techniques that were used are outlined below.

#### 2.3.1 Homology-based inference via BLAST

To develop the BLAST (Altschul *et al.*, 1990) homology-based predictor tool, the NCBI BLAST+ toolkit (version 2.11.0) was used. First, a target database was built from the training set using the makeblastdb command. The testing set was used as the query and searched against the target database using the blastp command. For the blastp search, the *e*-value cutoff was set at 1*e* + 6. This was done in order to obtain hits, even insignificant ones, for all the sequences in the testing set.

Once all the possible hits were obtained for all the sequences in the testing set, they were analysed to find the most significant hit. For each sequence in the testing set, this was done by first finding the hits with the lowest *e*-value. Among the hits with the lowest *e*-value, the hit with the greatest per cent sequence identity was chosen. This hit was deemed to be the prediction made by the model. This homology-based inference (HBI) via BLAST model is dubbed as the ‘BLAST model’.

#### 2.3.2 Artificial neural network

A simple artificial neural network (ANN) was developed for the task of remote homologue detection. The ANN had one hidden layer consisting of 128 nodes. The hidden layer was followed by the output layer. To reduce overfitting, a dropout (Srivastava *et al.*, 2014) layer with a rate of 0.3 and a batch normalization (Ioffe and Szegedy, 2015) layer were added to the model. The hidden layer used a rectified linear unit (Agarap, 2019) activation function, whereas the output layer used a Softmax (Bridle, 1989) activation function. The Adam (Kingma and Ba, 2017) optimizer was used with an initial learning rate of 1*e*−3. The model was set to train for a maximum of 200 epochs, but early stopping was implemented to prevent the model from overfitting. Early stopping was measured on the validation accuracy with the patience set to 20 epochs. A batch size of 256 was used for the training process. Three ANNs were trained independently on three different features, that is, ProtBERT embeddings, ProtT5 embeddings and Protein Sequence Lengths, to make the ‘ANN + ProtBERT’, ‘CATHe’ (the ANN model trained on the ProtT5 embeddings) and ‘ANN + Length’ models, respectively.

The significance of the ‘ANN + Length’ model comes from the assumption that the protein sequence embeddings generated by the pLMs, though they were of fixed size, were able to distinguish between small and long sequences. This model was developed in order to make sure that the classification of the protein sequences into CATH superfamilies was not simply based on their respective sequence lengths.

To arrive at the architecture that we use for the ANN, we conducted an optimization study where we measured the performance of different ANN architectures trained on the ProtT5 embeddings on the testing set of TOP 1773 SUPERFAMILIES, and TOP 50 SUPERFAMILIES datasets. We noticed that the model outlined here performed at par with more complex ANN models and that models with fewer parameters greatly underperformed, hence we chose this architecture (refer to Supplementary Section S2.2).

#### 2.3.3 Logistic regression

Logistic regression (LR) is a traditional machine learning method that is often used as a baseline to put the performance of more complex ANN models into perspective. In this study, we developed an LR model using the SciKit-Learn (Pedregosa *et al.*, 2011) python library. An ‘lgbfs’ solver was used with the maximum iterations set to 5000. The other parameters were set to the default values. This LR model was trained on the ProtBERT and ProtT5 embeddings to make the LR + ProtBERT and LR + ProtT5 models, respectively.

#### 2.3.4 Random

The random predictor model assigns an output class for each of the sequences at random taking into account the class imbalance. This serves as an important sanity check for measuring and comparing the performance of the proposed model.

**Fig. 1.**
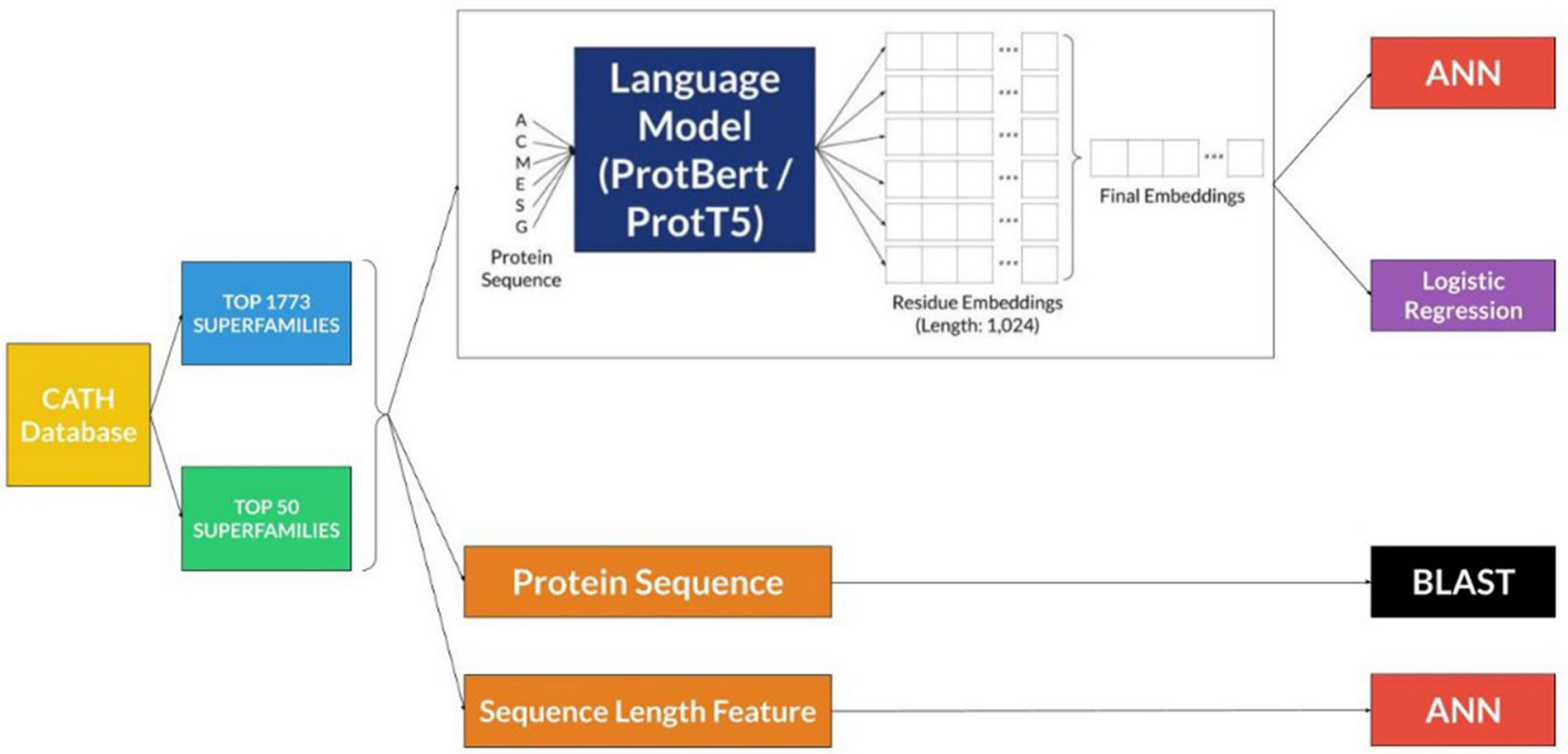
Illustration of the pipeline followed in this study which includes the generation of the two datasets, TOP 1773 SUPERFAMILIES and the TOP 50 SUPERFAMILIES, the features extracted from these datasets and the various models that were developed

In Figure 1, we outline the data preprocessing steps and the various models that we have used in this study in order to develop a tool for the detection of remote homologues in the CATH database. Supplementary Table S7 has a summary of the seven different models developed in this study.

### 2.4 Metrics

Four different metrics, accuracy, F1-score, MCC and balanced accuracy, were used to measure the performance of the models in this study. Refer to Supplementary Section S2.3 for more information on the metrics and how they are calculated.

### 2.5 Validation of CATHe by structure-based analysis of Pfam domains assigned to CATH superfamililes by CATHe

#### 2.5.1 Generation of Pfam domain set

In order to test if CATHe can detect remote homologues, we generated a set of Pfam domains that do not have annotations in the CATH database, that is, could not be detected by CATH-HMMs. The CATH-HMMs are derived for CATH S95 representatives (by running HMMER3 on all non-redundant representative sequences at 95% sequence identity from each of the CATH superfamilies). To generate the Pfam set of domains missed by CATH-HMMs, we scanned UniProt protein sequences (version 2019_05) against CATH-HMMs (derived from CATH v 4.3). Approximately 60% of the UniProt predicted domains can be assigned to CATH superfamilies using CATH-HMMs. Any regions not matching the CATH-HMMs were scanned against the library of Pfam-HMMs (from Pfam v 33.1). Those that matched Pfam-HMMs were used to compile our Pfam domain test set and the CATHe model trained on the TOP 1773 SUPERFAMILIES dataset was used to determine whether these could be assigned to CATH superfamilies based on CATHe predictions.

#### 2.5.2 Pfam-human domains

There are a total of 36 318 Pfam domains from *H.sapiens* which are not in CATH (Pfam v33.1 and UniProt 2019_05 were used for the generation of this dataset) but for which AF2 has predicted structures. In order to study the performance of CATHe and structurally validate the CATHe CATH predictions for these domains, we decided to choose only those domains for which the AlphaFold structure did not have any structurally problematic regions. By problematic, we mean domains with regions that will make it harder to detect structural relationships with CATH structural domains. The Pfam domain structures were obtained from the EBI AF2 database (https://alphafold.ebi.ac.uk/) (Jumper *et al.*, 2021; Varadi *et al.*, 2022). An AF2 domain was considered problematic if it met any of the following conditions:

- Model quality (pLDDT) is less than 90%.
- Less than three secondary structures in the 3D structure of the domain. This was calculated using DDSP (Kabsch and Sander, 1983).
- The longest problematic region (the longest stretch of residues that had less than 70% pLDDT) covered more than one-third of the domain length.
- Disorder greater than 70%. This was also measured using DSSP. The residues for which the DSSP secondary structure is defined by ‘-’ (None) was considered to be disordered.

Of these Pfam domains with good quality 3D models not mapping to CATH, CATHe predicted 197 domains with a good prediction probability (probability threshold of 90%; 0.5% error rate). This domain set is named ‘Pfam-human’.

#### 2.5.3 SSAP threshold analysis and expansion of CATH superfamily structural data using AF2 structures

The domains that are assigned to CATH superfamilies using CATH-HMMs or CATHe predictor were structurally validated by comparing their AF2-predicted structure against structural relatives in the predicted superfamily using SSAP (Taylor and Orengo, 1989). Specific SSAP score thresholds were applied for the different CATH protein classes (i.e. score thresholds of 71, 66 and 69 for CATH classes alpha, beta and alpha–beta, respectively). We also applied a structural domain overlap threshold of 60%. These thresholds are associated with a 5% error rate (see Supplementary Section S2.5 for description of how the thresholds were determined).

The addition of AF2 structures from the CATH-HMM matches greatly expands the structural data in CATH (by ∼60% on average).

#### 2.5.4 Comparison of human domains from CATH-HMM matches and CATHe matches

In order to gauge how remote the CATHe assigned *Pfam-human* domains are relative to human domains assigned to CATH superfamilies by the CATH-HMMs, we compared these two sets in terms of the SSAP scores between their AF2 domains and the closest relative in the CATH superfamily to which they were assigned. For both CATHe and CATH-HMM, we only considered comparisons involving good quality AF2 structures. For CATHe, this filtering left 150 domains (out of 197); for CATH-HMMs, this left 14 790 domains (out of 39 405) where the structural domain overlap from SSAP was greater than 60%.

#### 2.5.5 Understanding disease mutations in *Pfam-human* domains using CATHe

We performed a case study on the *Pfam-human* domains and studied mutations in them which could possibly be associated with diseases. In order to assess the likely impact of mutations in these domains, we analyse their proximity to conserved sites in the domain families. Therefore, the *Pfam-human* domains were aligned with sequences in their CATHe-predicted superfamily using jackhmmer (Johnson *et al.*, 2010) (with an *e*-value cutoff of 1*e*−5, set for three iterations). In order to detect conserved residues we used the scorecons (Valdar, 2002) tool and identified domains for which the alignments had a diversity of positions score greater than 80, reflecting an alignment informative enough to detect conserved residues. A scorecons score cutoff of 0.7 was then used to detect conserved residues.

## 3 Results

To develop our novel machine learning model for homologue detection, we generated a training set with CATH domains (version 4.3) (Sillitoe *et al.*, 2021) from the whole database (i.e. CATH relatives with known structure from PDB and those with predicted CATH structural annotations, from UniProt), whereas the testing and validation sets consisted only of domains of known structure in CATH. To be certain that the models learned to detect remote homologues, we ensured there was less than 20% sequence identity between and within the three sets.

To convert the domains into a format accessible for the machine learning models, we generated sequence embeddings for all of them. The performance of the proposed CATHe model (which is an ANN model trained on ProtT5 embeddings) was compared with six other baseline models: (1) HBI via BLAST (Altschul *et al.*, 1990), (2) an ANN trained on ProtBERT (Elnaggar *et al.*, 2022) embeddings, (3) an ANN trained only on sequence lengths, (4) LR trained on ProtBERT embeddings, (5) LR trained on ProtT5 embeddings and (6) a random baseline (refer to Section 2.4 and Supplementary Table S7). The performance of CATHe and the six other baseline models was measured using four metrics: accuracy, F1-score, MCC and balanced accuracy (refer to Supplementary Section S2.3).

### 3.1 Evaluation on the TOP 1773 SUPERFAMILIES dataset

The TOP 1773 SUPERFAMILIES dataset consisted of 1773 individual, well-populated (≥1 sequences) CATH superfamilies and an additional ‘mixed-bag’ set called the Other superfamilies (refer to Section 2.1). This set includes all CATH domain superfamilies containing a sufficient number of sequences (≥1) for testing and validating each.

**Fig. 2.**
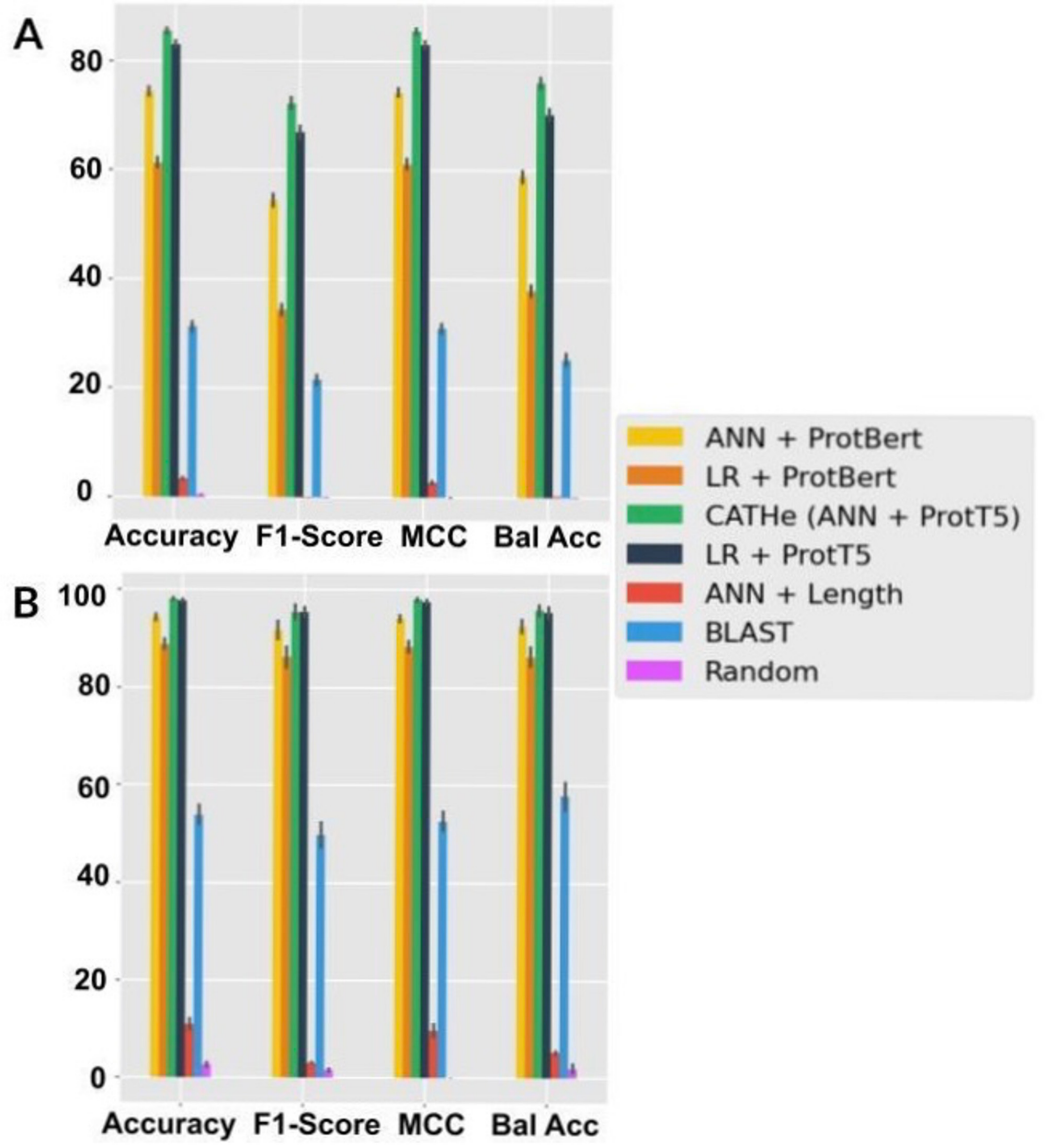
(**A**) Performance comparison using TOP 1773 SUPERFAMILIES. Seven methods for remote homology detection for CATH homologous superfamilies were compared using the TOP 1773 SUPERFAMILIES test set: two models trained on ProtT5 embeddings (ANN and LR with the ANN being dubbed CATHe), two models trained on ProtBERT embeddings (ANN and LR) and three baselines consisting of HBI via BLAST, an ANN trained only on protein sequence lengths and a random baseline. The performance was measured using accuracy, F1-score, MCC and balanced accuracy (Bal Acc) along with 95% confidence intervals. The proposed model, CATHe, has the highest performance and the machine learning models that were trained on pLM embeddings outperformed BLAST. (**B**) Performance comparison using TOP 50 SUPERFAMILIES dataset, the previously mentioned approach with the seven methods and four metrics were used in this analysis as well. We notice a similar trend that the proposed CATHe model had the highest performance and the machine learning models trained on pLM embeddings had a better performance than the baselines (including BLAST)

The performance of CATHe and the six other baseline models was measured on the testing set of the TOP 1773 SUPERFAMILIES dataset. The results are highlighted in Figure 2(A) and described in more detail in Supplementary Table S1(A). The performances are given along with a 95% confidence interval obtained by conducting bootstrapping on the testing set with 1000-folds. The standard deviation obtained from this bootstrapping method was multiplied with 1.96 to obtain the 95% confidence interval values. When comparing these methods, we noticed that the two models trained on ProtT5 embeddings (CATHe and LR + ProtT5) outperformed all other methods, including those trained on ProtBERT (ANN + ProtBERT and LR + ProtBERT). Among models trained on ProtT5, the ANN outperformed the LR with an F1-score of 72.4 ± 0.7%. Compared with these embedding-based approaches, BLAST reached lower performance but outperformed the ANN + Length model which improved only slightly over the random baseline. In general, we can see that the models trained on embeddings from pLMs outperformed traditional methods like BLAST.

### 3.2 Evaluation on the largest 50 superfamilies

The TOP 50 SUPERFAMILIES dataset is a subset of the TOP 1773 SUPERFAMILIES dataset, consisting only of the 50 largest CATH superfamilies chosen according to the population of the superfamily in the TOP 1773 SUPERFAMILIES dataset (refer to Section 2.2). These are highly populated superfamilies accounting for 37.32% of all non-redundant domains in CATH. We re-trained CATHe and the six baseline models and measured their performance on the testing set for the TOP 50 SUPERFAMILIES dataset using the four performance metrics. The results are highlighted in Figure 2(B) and described in more detail in Supplementary Table S1(B).

Again, the highest performance (F1-score of 95.5 ± 0.9%) among all these seven models was achieved by CATHe. The performance of the other six models follows a similar trend as for the TOP 1773 SUPERFAMILIES dataset, that is, models trained on ProtT5 embeddings outperform models trained on ProtBERT embeddings and pLM-based methods outperform baselines, including BLAST. Furthermore, the CATHe superfamily predictions for this test set were the same for the TOP 50 SUPERFAMILIES model and the TOP 1773 SUPERFAMILIES model.

In an effort to check what CATHe is learning and to understand to what extent CATHe captured superfamily-specific information during training, we compared the original ProtT5 embeddings of the protein domain sequences and the embeddings derived from the final layer of the CATHe ANN after training (dubbed CATHe embeddings). t-SNE (van der Maaten and Hinton, 2008) was used to project the high-dimensional embeddings, for both the ProtT5 (which were 1024-D) and CATHe embeddings (which were 128-D), in 2D.

**Fig. 3.**
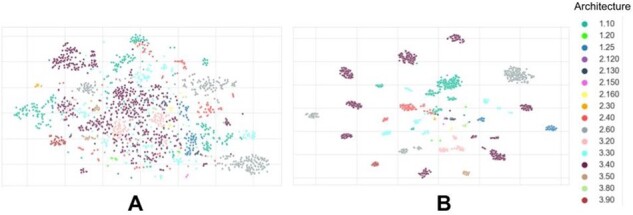
t-SNE projections of high-dimensional embedding spaces for the TOP 50 SUPERFAMILIES test set coloured by CATH architectures. (**A**) The information learned by the pLM ProtT5 during self-supervised pre-training, that is, without any supervised training. (**B**) Embeddings were extracted from the last layer of the trained CATHe ANN to highlight the additional benefit of supervised training for distinguishing CATH architectures

Colouring the 2D projections (Fig. 3A) based on CATH domain architecture (i.e. the arrangement of secondary structures in 3D) shows that embeddings from the ProtT5 pLM are able to cluster some of the architectures such as 2.60, 2.40 and 1.10 quite well, but the larger, more diverse architectures such as 3.40 and 3.30 are more dispersed. This is likely to be due to the fact that there are many different topologies for the superfamilies in these latter two architectures (126 and 224 topologies present in the 3.40 and 3.30 architectures, respectively, compared with <50 topologies for most of the remaining architectures). Furthermore, these latter two architectures comprise superfamilies that are particularly structurally diverse, in some cases showing 3-fold or more variation in the size of the relatives (Dessailly *et al.*, 2010) (refer to Supplementary Figs S3 and S4).

The distinct clustering of CATH architectures in CATHe embedding space (Fig. 3B) indicates that CATHe is able to recognize differentiating information about the domain structure architecture level during the training process. Additionally, we notice that CATHe is also capable of recognizing fold-level information. 2D t-SNE plots were generated for the different topologies in the 3.40 architecture and we notice that the point clouds for the different topologies in this architecture are well separated (refer to Supplementary Fig. S5).

### 3.3 Checking for class imbalance

A number of tests were conducted to determine how the class imbalance for specific homologous superfamilies in the two datasets, TOP 1773 SUPERFAMILIES and the TOP 50 SUPERFAMILIES, affected the CATHe performance. We examined the relationship between the population of a superfamily as defined by the number of sequences for this superfamily in the training set and the CATHe performance for these superfamilies in terms of F1-score, but found no linear relationship (see Supplementary Figs S1A–C and S2A–C). Further analysis of the relationship between the CATHe performance for a superfamily and the structural diversity present in the superfamily as defined by the number of structurally similar groups at 5Å (SSG5 groups) also failed to detect a direct linear relationship (Supplementary Figs S1D and S2D). These studies are discussed in more detail in Supplementary Section S1.1.

### 3.4 Further evaluation of CATHe by comparison against HMMs

We considered directly comparing the performance of CATHe against state-of-the-art HMM-based techniques [e.g. HMMer (Mistry *et al.*, 2013)]. However, conducting a direct comparison between the performance of these two techniques on the same dataset would mean building HMM libraries from the sequences of training sets of the TOP 1773 SUPERFAMILIES and TOP 50 SUPERFAMILIES datasets. This could result in poorly performing HMMs as the alignments for building the HMMs would be derived from very small sequence sets (i.e. non-redundant sets at <20% sequence identity) making it challenging to perform a direct and fair comparison.

Therefore, we compared the HMM and CATHe performance in an indirect fashion. We determined whether CATHe was able to assign any Pfam domains in UniProt sequences missed by scanning the sequences against CATH-HMMs for CATH superfamilies. There are nearly 38M domain regions in sequences from UniProt [version 2018_02 (UniProt Consortium, 2015)] which do not map to CATH-HMMs but which can be mapped to Pfam (see Section 2.5). To obtain a more tractable number for our CATHe evaluations, we clustered these at 60% sequence identity (to ensure significant structural and functional similarities) using MMseqs2 (Radivojac *et al.*, 2013).

The resulting set (named *PFAM-S60*) had 10.5M domains which were converted to embeddings using ProtT5. In order to obtain a reliable threshold on prediction probability for our CATHe model, we measured the error rate at various prediction probability thresholds and concluded that by only allowing predictions with a prediction probability above 0.9 (or 90%, which corresponds to a 50% coverage), we ensure an expected error rate of 0.5% (for additional details see Supplementary Section S2.4).

These CATHe assignments were then validated by structure comparison. Although these domains do not have experimental structures in the PDB, where available we used good quality 3D-models from the AF2 dataset at the EBI (Jumper *et al.*, 2021; Varadi *et al.*, 2022) for the structural validation. Since assignment of very remote homologues to CATH also involves manual inspection of the match, we selected a tractable number of domains and chose a subset of the *Pfam-S60* set belonging to *H.sapiens*. Allowing an error rate of 0.5% and only testing sequences with AF2 models of good quality (see Section 2.5) CATHe predicted 197 domains with a good prediction probability (named *Pfam-human*).

To structurally validate the CATHe superfamily predictions for *Pfam-human*, we used our in-house protein structure comparison method, SSAP (Taylor and Orengo, 1989) to compare them against structural relatives in the CATH superfamily to which they were assigned by CATHe. However, since the *Pfam-human* domains are likely to be very remote homologues of CATH superfamilies, the CATH superfamilies were first expanded (by ∼60%, on average) by including AF2-predicted structural regions corresponding to close CATH-HMM matches already present in the CATH database (see Section 2.5). The SSAP thresholds for a structural match were set at a SSAP score of 71, 66 and 69 for CATH classes alpha, beta and alpha–beta, respectively, with an additional threshold of 60% for the structural domain overlap (see Section 2.5).

**Fig. 4.**
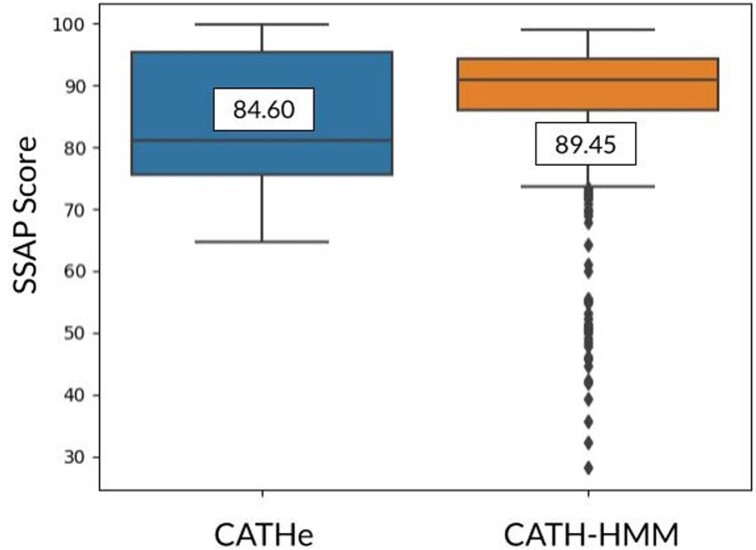
Comparison of SSAP scores for superfamily predictions made by the CATH-HMMs for Gene3D domains from *H.sapiens* (orange box) and CATHe for Pfam domains from *H.sapiens* (blue box)*.* The SSAP boxplots for both of these sets have been annotated with their average SSAP score. The CATH-HMM set had a greater average SSAP score than the *Pfam-human* set showing that they tend to be closer homologues to the existing CATH superfamily relatives than those detected by CATHe

We could structurally validate 142 out of the 197 of the *Pfam-human* domains predicted to belong to a specific CATH superfamily by CATHe. Additionally, manual curation on the 55 domains that did not cross the SSAP thresholds confirmed that 37 more domains were valid superfamily matches (refer to Supplementary Section S1.1 for more information on the manual curation). Therefore, a total of 179 domains out of 197 (90.86%) from *Pfam-human* that matched CATH superfamilies using CATHe could be structurally validated as true positives. This experiment confirms our hypothesis that although CATHe was trained on the CATH-HMM-predicted domains, it goes beyond this set of relatively close homologues to detect more remote homologues. Further analyses showed that domains assigned by CATH-HMM had a greater average SSAP score (89.45) than the CATHe set of human domains (84.60) (Fig. 4) (see Section 2.5 for further details).

#### 3.4.1 Assessing the value of the human Pfam assignments and assigning further Pfam domains to CATH

**Fig. 5.**
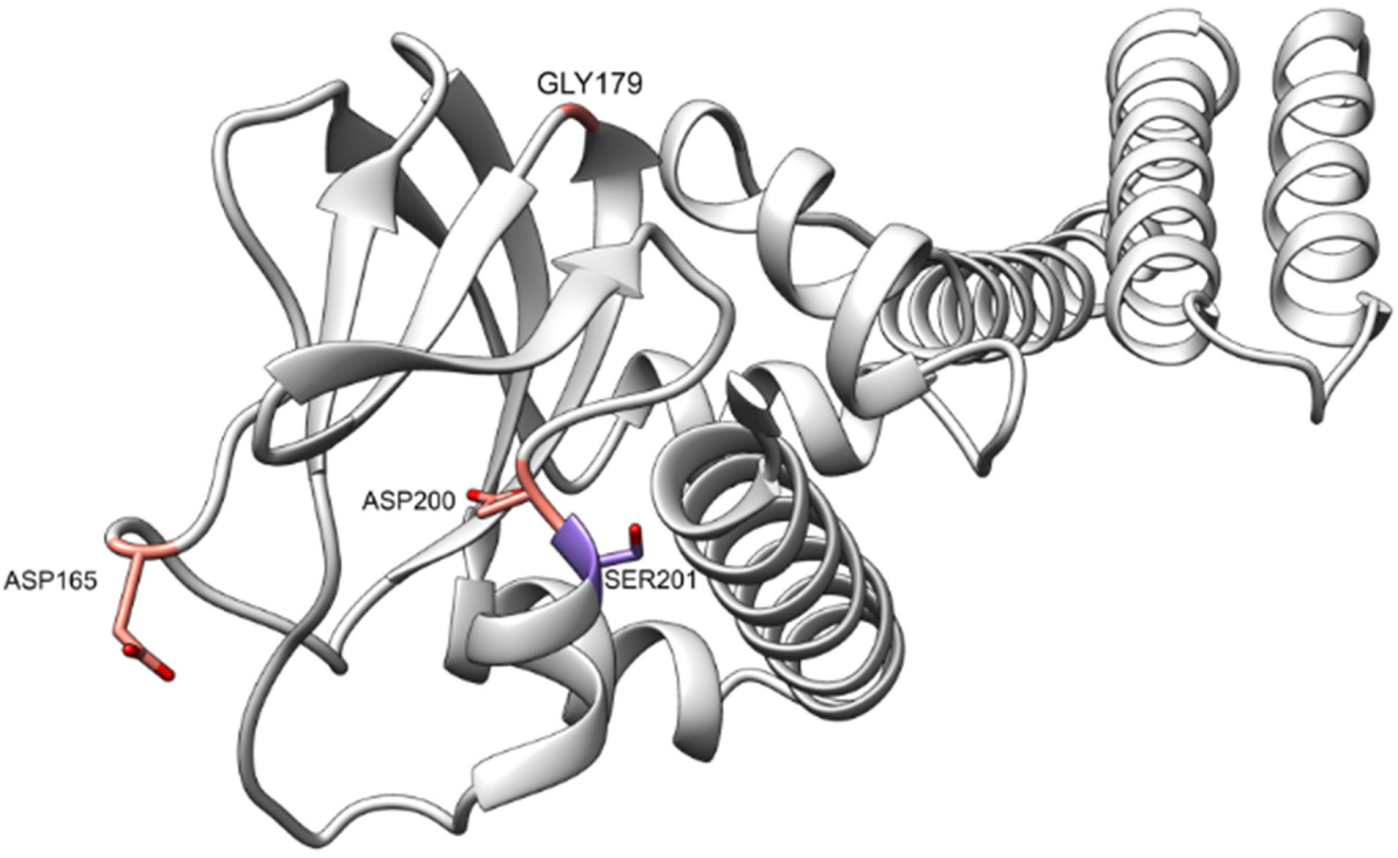
AF2-predicted structure of Q8NE79 blood vessel epicardial substance protein for the residue range 40–267. In this structure, the conserved residues: ASP 165, GLY 179 and ASP 200 are coloured in red whereas the residue with the disease associated mutation SER 201 is coloured in purple

We analysed all of the CATHe predictions in the *Pfam-human* set to assess the value of these new assignments and noticed that there are four domains with possible disease causing mutations. For one of these ‘Q8NE79_40_267’ (Q8NE79: Blood vessel epicardial substance protein—residue range, 40–267), we observed that the disease associated mutations were close to conserved residues (See Section 2.5 for details). This domain was assigned to the CATH superfamily 2.60.120.10 by CATHe with a 100% prediction probability and was subsequently manually curated to confirm the superfamily match. Residues 165, 179 and 200 were found to be conserved [scorecons (Valdar, 2002) score > 0.8]. We observed that there is a mutation in residue 201, Ser201Phe, which lies near highly conserved residues, possibly causing a functional impact which leads to limb-girdle muscular dystrophy autosomal recessive 25. Serine 201 is well conserved with a scorecons value of 0.786. The AF2 structure with the conserved and mutated residues in this domain is depicted in Figure 5.

#### 3.4.2 CATH superfamily expansion using CATHe

Since our analyses with the *Pfam-human* subset showed that the CATHe predictions are highly reliable (we were able to structurally validate 90.86%), we used CATHe to predict the superfamily annotations for the full *Pfam-S60* set which contained 10.5M domains. Applying the threshold probability of 0.9 to CATHe predictions for the *PFAM-S60* set allowed us to assign CATH homologous superfamilies to roughly 4.6M (4 623 730) *PFAM-S60* domains (43.72% of the *PFAM-S60* set). Adding these newly predicted domains to CATH v 4.3 led to a 3.06% increase in database size.

## 4 Discussion and conclusion

The CATH database currently uses a combination of protein structure comparison and sequence comparison tools to identify homologues for CATH superfamilies (Sillitoe *et al.*, 2021). In this study, we present a deep learning-based tool, CATHe, that trains an ANN on embeddings from the pLM ProtT5 to detect remote homologues for these CATH superfamilies. Our models perform well—an accuracy of 85.6 ± 0.4% for the model based on a large set of 1773 CATH superfamilies and an accuracy of 98.2 ± 0.3% for the model based on the 50 most highly populated CATH superfamilies.

We set very stringent levels of non-redundancy between the training and test sets to make sure that we have a test dataset that properly encapsulates the features of remote homologues. This threshold (≤20% sequence identity) was much stricter than applied in related studies on protein sequence classification such as DeepFam (Seo *et al.*, 2018) and DeepNOG (Feldbauer *et al.*, 2020) which did not apply any redundancy removal filters, and Bepler and Berger’s (2021) study that used a sequence identity filter of 40%, but CATHe achieved a comparable performance, and higher performance for the TOP 50 SUPERFAMILIES model.

We further validated the performance of CATHe and demonstrated that it could outperform HMM-based protocols for homologue detection by using CATHe to predict CATH superfamilies for *Pfam-human* domains not detected by CATH-HMMs. Validation of these CATHe predictions by structure comparison of the AF2-predicted structures against CATH structures gave an accuracy of 90.86%. We consider this a compelling result as Pfam domains do not always correspond to a single domain, they can comprise partial or multiple domains making it harder for CATHe to detect the relationship.

Less than 20% of CATH superfamily relatives have structural characterization and these may be extremely remote, structurally diverse homologues of the *Pfam-human* domains. In some CATH superfamilies, structural similarity between very distant homologues can fall below 50%. Therefore, it is possible that more of the CATHe predictions are correct. In fact, manual evaluation of those not verified by structure comparison showed that most (∼90%) had the correct architecture (i.e. arrangement of secondary structures in 3D), suggesting CATHe is able to capture important elements of the structural environment.

Although the CATHe model made a total of 36 misclassifications on the testing set of the TOP 50 SUPERFAMILIES dataset (see Supplementary Table S2), manual analysis showed that even when the CATHe-predicted superfamily annotation was incorrect, CATHe was able to correctly predict the Class (level 1 of CATH hierarchy) and Architecture (level 2 of CATH hierarchy) for 23 and 14 domains, respectively. This again suggests that CATHe is able to capture elements of secondary structures and the 3D packing of secondary structures (refer to Supplementary Section S1.1 for more information).

For both datasets (TOP 1773 SUPERFAMILIES and TOP 50 SUPERFAMILIES), we found that the ANN models (CATHe and ANN + ProtBERT) outperform the two LR models (LR + ProtT5 and LR + ProtBERT), justifying the usage of an ANN which has a higher number of parameters when compared with a LR model. However, for the TOP 50 SUPERFAMILIES dataset, the increase in performance relative to the LR + ProtT5 models is not statistically significant, most likely due to the shallow nature of the neural network used in CATHe. This could mean that both CATHe and the LR model learn similar representations of the data resulting in similar performances. It has already been shown previously that even very simplistic models achieve competitive performance when using ProtT5 embeddings as input. This is in line with results from NLP where simple networks applied together with embeddings achieve competitive performance. One working hypothesis is that the LM already extracts very informative embeddings that are easily ‘readable’ by downstream predictors. So, the heavy lifting of feature extraction is already done by the LM. Vice versa it is difficult to improve over this result with more complex networks as it might be difficult to extract additional information that was not already extracted by the pLM, especially, given the limited dataset size of most supervised tasks. We refer to these publications for more details and discussion (Elnaggar *et al.*, 2022; Ilzhöfer *et al.*, 2022).

The models trained on the ProtT5 embeddings (CATHe and LR + ProtT5) perform better than the models trained on the ProtBERT embeddings (ANN + ProtBERT and LR + ProtBERT), this was noticed in the initial study of these pLMs (Elnaggar *et al.*, 2022). As regards the performance of ProtT5 relative to ProtBERT—one of the most striking differences between the two is model size: ProtT5 has a total of 3B parameters (1.5B for the encoder that is used here) while ProtBERT has only 420M. This gives ProtT5 much more capacity to absorb information. While there is clearly a limit on just scaling model size up, for example, the larger ProtT5-XXL with 11B parameters performed worse than its 3B pendant used here, this limit seems not to be reached by the comparatively small ProtBERT.

Furthermore, from the low performance of the ANN + Length, we conclude that sequence length is not a feature that is very useful for superfamily classification. The significant improvement in the performance of the ANN models trained on sequence embeddings (CATHe and ANN + ProtBERT) over ANN + Length suggests that these models not only capture sequence features such as length but are also able to extract superfamily-specific information from the sequence.

A limitation of our method is the number of sequences needed for training the models which means that we cannot currently classify distant homologues for all the 5481 superfamilies in CATH. The models we built using highly populated superfamilies (TOP 50 SUPERFAMILIES dataset) significantly outperformed the model trained on superfamilies having far fewer sequences (98.2% on the TOP 50 SUPERFAMILIES dataset and 85.6% on the TOP 1773 SUPERFAMILIES dataset). However, all the publicly available sequence repositories [e.g. UniProt (UniProt Consortium, 2015) and Mgnify (Mitchell *et al.*, 2020)] are expected to continue their exponential increases in the foreseeable future and the significant expansion of predicted structural data by AF2 and related methods (Lin *et al.*, 2022; Wu *et al.*, 2022) will significantly expand the populations of the CATH superfamilies, to enable training of more powerful CATHe models that can classify homologues for all CATH superfamilies.

Our models were trained using highly non-redundant datasets to evaluate the performance for detecting very remote homologues. In the future, we will also train our models using the full sequence datasets available for the superfamilies so that we can apply CATHe to bring in closer homologues as well. Since CATHe is at least twice as fast as our CATH-HMM-based protocol, new classification pipelines for the CATH database will employ these faster and more sensitive sequence embeddings-based approaches. More sensitive sequence-based homologue detection tools than HMMs (e.g. HMM–HMM-based methods) may outperform pLMs, like CATHe, for remote homologue detection, but these are even slower than HMMs.

Other protein family resources [e.g. Pfam (Mistry *et al.*, 2021) and InterProscan (Mitchell *et al.*, 2019)] are also considering classification protocols that exploit these faster, more energy efficient and therefore more environmentally sustainable approaches. We will work with those communities to evaluate the best strategies and monitor improvements in performance.

In summary, our method was tested on a dataset of very remote homologues (less than 20% sequence identity). To our knowledge, it is the only method that has been evaluated on such a strict dataset and subsequently validated by structural comparison using AF2 3D models for predicted classifications. We demonstrated that 4.62 million Pfam domains previously unmapped to CATH could be brought in using a reliable threshold on accuracy and error rate (0.5% error rate). This will expand CATH by 3.06%. However, Pfam does not classify all of UniProt and we will apply CATHe in the future to detect additional domains in UniProt, not classified in Pfam, that can be assigned to CATH superfamilies. Where possible, we will use good-quality AF2 structures to confirm these assignments. The CATH domain sequence embeddings generated by ProtT5 and the source code for the CATHe model can be found at https://github.com/vam-sin/CATHe.

## Supplementary Material

btad029_Supplementary_Data
